# A framework for community ownership of a text messaging programme to improve adherence to antiretroviral therapy and client-provider communication: a mixed methods study

**DOI:** 10.1186/1472-6963-14-441

**Published:** 2014-09-26

**Authors:** Lawrence Mbuagbaw, Renee-Cecile Bonono-Momnougui, Lehana Thabane, Charles Kouanfack, Marek Smieja, Pierre Ongolo-Zogo

**Affiliations:** Department of Clinical Epidemiology and Biostatistics, McMaster University, Hamilton, ON Canada; Biostatistics Unit, Father Sean O’Sullivan Research Centre, St Joseph’s Healthcare—Hamilton, Hamilton, ON Canada; Centre for Development of Best Practices in Health, Yaoundé Central Hospital, Yaoundé, Cameroon; Departments of Paediatrics and Anaesthesia, McMaster University, Hamilton, ON Canada; Centre for Evaluation of Medicine, St Joseph’s Healthcare—Hamilton, Hamilton, ON Canada; Population Health Research Institute, Hamilton Health Sciences, Hamilton, ON Canada; Yaoundé Central Hospital Accredited Treatment Centre, Yaoundé, Cameroon; Faculty of Medicine and Biomedical Sciences, University of Yaoundé 1, Yaoundé, Cameroon; St. Joseph’s Healthcare Hamilton, Hamilton, ON Canada

**Keywords:** Text messaging, Community ownership, HIV, Mixed methods, Cameroon

## Abstract

**Background:**

Mobile phone text messaging has been shown to improve adherence to antiretroviral therapy and to improve communication between patients and health care workers. It is unclear which strategies are most appropriate for scaling up text messaging programmes. We sought to investigate acceptability and readiness for ownership (community members designing, sending and receiving text messages) of a text message programme among a community of clients living with human immunodeficiency virus (HIV) in Yaoundé, Cameroon and to develop a framework for implementation.

**Methods:**

We used the mixed-methods sequential exploratory design. In the qualitative strand we conducted 7 focus group discussions (57 participants) to elicit themes related to acceptability and readiness. In the quantitative strand we explored the generalizability of these themes in a survey of 420 clients. Qualitative and quantitative data were merged to generate meta-inferences.

**Results:**

Both qualitative and quantitative strands showed high levels of acceptability and readiness despite low rates of participation in other community-led projects. In the qualitative strand, compared to the quantitative strand, more potential service users were willing to pay for a text messaging service, preferred participation of health personnel in managing the project and preferred that the project be based in the hospital rather than in the community. Some of the limitations identified to implementing a community-owned project were lack of management skills in the community, financial, technical and literacy challenges. Participants who were willing to pay were more likely to find the project acceptable and expressed positive feelings about community readiness to own a text messaging project.

**Conclusion:**

Community ownership of a text messaging programme is acceptable to the community of clients at the Yaoundé Central Hospital. Our framework for implementation includes components for community members who take on roles as services users (demonstrating clear benefits, allowing a trial period and ensuring high levels of confidentiality) or service providers (training in project management and securing sustainable funding). Such a project can be evaluated using participation rate, clinical outcomes, satisfaction with the service, cost and feedback from users.

**Electronic supplementary material:**

The online version of this article (doi:10.1186/1472-6963-14-441) contains supplementary material, which is available to authorized users.

## Background

Worldwide, more than 35 million people are living with human immunodeficiency virus (HIV) [1]. Despite the increasing numbers of people on life-saving antiretroviral therapy (ART), millions of people continue to suffer from the morbidity and mortality associated with HIV [1]. In low-and middle-income countries only 34% of eligible individuals are actually receiving ART. Among those who are receiving ART, suboptimal levels of adherence to medication often occur and are a major challenge to HIV care [2]. Considerably high levels of adherence are required to suppress viral replication and boost CD4-positive-T-lymphocyte count [3–6]. The consequences of poor adherence to ART include not only accrued morbidity and mortality, but also higher transmission rates, the development of resistant viral strains [3–6] and overall increments in health care costs. Adherence to medication, generally speaking, and specifically for ART is linked to multiple factors which may be related to the patient, provider, health system or the medication itself [7]. The WHO encourages multi-faceted adherence improvement initiatives that are tailored to patients’ needs [1, 7].

In recent years, text messaging has emerged as an effective supporting tool for adherence to ART [1, 8–10]. Currently, there is ongoing research on how best to scale-up text messaging interventions and how to tailor them to suit patient characteristics [11].

About 70% of people living with HIV (PLHIV) reside in sub-Saharan Africa [12]. Among the West and Central African countries, Cameroon has one of the highest prevalence of HIV, with 4.6% of the adult population infected [12]. The situation is worsened by sub-optimal adherence to antiretroviral medication and a fragile health system plagued by human resource shortages and medication stock-outs [13]. An impressive mobile phone penetration rate of 52% [14] and affordable cellular communication offer an opportunity to resolve some of these difficulties through mobile-health (m-Health) interventions.

Community involvement is a frequent characteristic of successful health care programmes [15] and can play an important role in improving adherence [1]. This is most likely because of the potential for better outreach and the possibility of mobilising community resources such as finances, man-power and material. We argue that communities have the right to take part in decisions affecting their lives and are in the best position to defend their own interests, therefore it is only wise that they be involved in developing community-run programmes [16].

This research builds on previous research findings, indicating the effectiveness of text messaging in improving adherence to ART, especially two-way weekly text messages [9, 17], and the potential of text messaging in resolving other unmet communication needs [18, 19]. There is also evidence for health worker support of text messaging programmes [20]. Putting the available evidence together suggests a favorable environment for scaling up text messaging interventions but leaves important knowledge gaps regarding how best to take text messaging programmes to scale and how to use text messaging to enhance communication and care in general. Owing to the fact that text messaging, though technology-based, is a relatively simple intervention, and community involvement comes with numerous advantages, we sought to investigate community willingness and readiness to own and run a text messaging programme using mixed-methods.

For the purposes of this study, “community” will refer to the group of individuals living with HIV and receiving care from the Yaoundé Central Hospital Accredited treatment Centre (YCHATC) and ownership as “as a process in which the community members design, manage and reap benefits from a programme” [16].

The purpose of this research is to identify key factors linked to community willingness and readiness to own a text messaging programme, and to use this information to develop a framework for transfer of text messaging-based initiatives to community groups.

### Research paradigm

We adopted a pragmatist paradigm to shape and design our research. In order to develop a framework for community ownership that is applicable in the real world, we used both deductive and inductive reasoning to determine what factors would enter the framework. Pragmatism has been argued to be a preferred paradigm for mixed-methods research as it allows the use of “what works” best in data collection and analysis, incorporates multiple perspectives and links subjective and objective knowledge [21–23]. In this paradigm we acknowledge that our research is occurring within specific social, economic and political contexts [24].

### Theoretical framework

Admittedly, text messaging for health care is not so new, as the medical literature is growing with examples of text messaging interventions with favorable outcomes in HIV care [8, 10, 18, 25–27]. However, community ownership of such, to the best of our knowledge, has not been described or investigated. The theory of “diffusion of innovations” suggests that innovation factors (relative advantages to be gained, compatibility with existing practices, simplicity of use, trialability and observable results) receiver factors (attitude to change, perceived need) and social system factors (social norms and tolerance for deviancy) will affect the adoption of community ownership of a text messaging programme. Adoption would occur in five steps: knowledge, persuasion, decision, implementation and confirmation. We sought to identify the roles of these factors and integrate them in the framework development process [28].

### Why mixed methods

Mixed methods are now being recognised as a strong research option for complex research questions that cannot be answered by either qualitative or quantitative methodologies applied singly [29]. Mixed methods can reap the advantages of breadth and generalizability obtained from quantitative methodologies, and also the depth in understanding phenomena from qualitative methodologies. Overall, mixed methods offer a more complete picture for a research question [30]. In this work, we sought to complement the qualitative with the quantitative, to initiate new ways of thinking and to expand existing knowledge [31]. We provided further justification for our use of mixed methods in the protocol [16].

### Research questions

This work was guided by qualitative, quantitative and mixed research questions [16].

#### Qualitative research questions

- Will PLHIV in Yaoundé, Cameroon accept community ownership of a text messaging programme and are they ready to take ownership?

#### Quantitative research questions

- How many of the PLHIV in Yaoundé, Cameroon are willing to accept and ready to own a text messaging programme and what factors are associated with acceptability and readiness?

#### Mixed methods research questions

- How generalizable are the themes related to acceptability and readiness to a larger sample of PLHIV in Yaoundé, Cameroon?
- What are the similarities and differences between the qualitative and quantitative phases?

## Methods

Our methods have been described in detail in a published protocol [16]. The essential portions are reported here.

### Ethics

Ethics approval was obtained from the Institutional Review Board (IRB) of the Yaoundé Central Hospital (N°288 L/MINSANTE/SG/DHCY/Stages on the 16 May 2013).

### Design and rationale for design

We employed the exploratory sequential design for this study. Simply put, it entails an initial qualitative strand that leads to thematic variables which are converted into questions for the second quantitative phase, after which the findings from both stands are merged and correlated to generate meta-inferences. This design enhances our ability to generalise qualitative findings, develop questions to measure community acceptability/readiness and to facilitate collaboration between researchers with qualitative and quantitative backgrounds [22]. It is also the most appropriate design for instances where there is no existing guiding framework [22]. Even though the exploratory sequential design is not the typical mixed methods design used in the pragmatic paradigm [24], it was the most appropriate for our purposes.

### Setting

This study was conducted in the Yaoundé Central Hospital Accredited HIV Treatment Centre (YCHATC), located in the capital city of the central African nation of Cameroon. It caters for the services of about 6500 clients and receives close to 40 new clients every week. Eight physicians provide daily care to clients. The services provided include screening for HIV, clinical care, psycho-social support, routine lab testing and hosting community associations of people living with HIV. In preceding years, this centre has been the focus of text messaging research [18, 20, 32]. The adult prevalence of HIV in Cameroon is 4.5% [1]. In the Cameroonian health system, most of the burden of health care expenditure (70.0%) is borne by out-of-pocket payments [33]. However, many costs are subsidized for vulnerable populations like children, pregnant women and people living with HIV.

### Sampling

We employed a two-stage sampling procedure, using purposive and probabilistic sampling techniques to ensure that data was collected in depth and breadth [34].

For the quantitative strand we sought individuals who were living with HIV, receiving care from the YCHATC or who fulfilled any of the following criteria: 1) they belonged to an association of PLHIV (as leaders or members), 2) they were community health workers, 3) they were willing to participate in a community owned text messaging programme. Participants with significantly different characteristics were interviewed separately to create homogenous groups. Focus groups of 6 to10 participants were conducted until saturation of ideas.

For the quantitative strand, we applied the sample size formula proposed by Cochran for surveys [35]. For a total population of PLHIV at the YCHATC of 6500, assuming an α level of 0.05, a 5% margin of error (for categorical data) and a standard deviation of 0.5 (for our binary primary outcome- willingness to participate in a community-led text messaging programme), after considering a 10% “refusal to participate” rate (obtained from a previous text messaging study in the same setting [36]) we arrived at a sample size of 402 participants [16].

### Data collection

In the qualitative strand, two teams of data collectors were formed, each made up of one moderator and one note taker, neither of whom were staff of the hospital. They were all dressed in plain clothes. During the daily information-education-communication (IEC) sessions held for clients of the YCHATC, clients were invited to take part in focus group discussions (FGDs). The consenting participants were invited into a separate room. Basic socio-demographic data were collected (age, gender and level of education), and participants were invited to pick a name they would wish to be addressed by during the discussions. The moderator introduced the session and initiated the discussions based on a focus group discussion guide [16]. All the discussions were recorded. The note taker also noted relevant parts of the discussion and important non-verbal cues that could not be captured by the audio recorder. The FGDs lasted about 60 minutes each. Participants were compensated for the time spent during the FGDs (1000 Frs CFA ~2 USD).

In the quantitative strand, clients in the waiting rooms of the YCHATC were approached and invited to take part in a survey. Our questionnaire (developed from the qualitative strand) was revised with the interviewers and pilot tested on ten participants for clarity. Four hundred and twenty nine clients were approached, of which nine declined to participate in the study. After oral consent was obtained, a questionnaire was administered. The questionnaire contained basic socio-demographic and clinical data, and questions derived from the themes which arose in the qualitative strand. The interviews lasted about 15 minutes each.

### Data analysis

We conducted a thematic analysis for the qualitative data. The notes taken were used to supplement the audio-recordings, which were transcribed into text. Codes were generated by identifying repetitions in the text [37]. Similar codes were grouped and themes generated from these groupings. Two coders worked on the data to verify agreement on the themes generated. In the case of disagreement, the final themes were determined by consensus. The themes were then converted into thematic variables and employed in the survey as questions. Quotes of illustrative significance are reported.

Quantitative data were analysed using Statistical Package for Social Sciences (SPSS) V.20.0 (SPSS, Inc, 2009, Chicago, Illinois, USA). Baseline characteristics and responses to questions are reported as counts (%) or mean (standard deviation [SD]). Community willingness to participate in a community-led text messaging programme was dichotomized and used as the dependent variable in logistic regression models. Independent variables such as age (years), gender (male or female), level of education (none, primary, secondary, university), profession (working, unemployed, retired, student), marital status (married or living together, single, divorced, widow or widower), residence, lodging situation (common or individual), time since diagnosis (months), duration on ART (months), self-reported adherence to medication (seven-point Likert scale), and self-reported adherence to appointments (seven-point Likert scale). The purpose of this adjusted analysis was to determine if there are any underlying factors which influence willingness to participate and which must be addressed in the development of a framework. The level of statistical significance was set at α = 0.05. Adjusted odds ratios (OR) are reported with 95% confidence intervals (CI). Model fit was estimated using Hosmer and Lemeshow’s goodness of fit statistic.

### Data integration

Patient characteristics and responses were compared numerically and graphically. In order to represent the merged data graphically we estimated confidence intervals around the proportions for key patient characteristics in both strands, themes (in the qualitative strand) and corresponding thematic variables (in the quantitative strand). We considered data convergence to exist if the confidence intervals overlapped in a forest plot. The confidence intervals were estimated for graphical purposes using the recommended formula based on standard errors [38]. This provides a graphical appraisal of comparability between samples with different denominators.

### Validation checks

The purpose and objective of this study were pre-specified in a published protocol [16]. In the qualitative strand, we used member-checking, audio recorders and duplicate coding to ensure the accuracy of our data. We limited facilitator bias by using a discussion guide. In the quantitative strand we estimated an appropriate sample size, revised and pilot-tested our questionnaire, and performed adjusted analyses to account for potential confounders.

### Team composition

Our research team at the Centre for Development of Best Practices in Health (CDBPH; http://www.cdbph.org) was constituted of a mix of researchers with expertise in both qualitative and quantitative research methods. We included public health physicians, anthropologists, sociologists, HIV clinical expert, experts in health policy and statisticians.

## Results

### Qualitative results

#### Participants

Seven focus groups discussions were conducted. Six of these were made of heterogeneous individuals with regards to age, gender, level of education and profession. The last focus group discussion included four leaders of HIV associations with a minimum of 11 years of experience with community activities. In total, 57 participants were included of which only 8 were male. The mean age (SD) was 38.8 (11.1). Close to half (54.3%) had a primary education, the rest had either secondary or university education. The strongest themes that emerged from these discussions were: weak participation in community activities, willingness to participate in a text messaging initiative and mixed feelings about community management.

#### Participation in community activities

Almost three-quarters of the participants did not participate in any community activities or belong to any specific community association. Such activities may include support groups, pill buddy groups, associations for lobbying or associations for home-based care. Some of them were not even aware that there were existing community associations. A few others described failed attempts at creating their own associations of people living with HIV. Generally speaking, participants felt they did not have sufficient information with regards to the functioning of community associations within and out of hospitals.

*“I don’t know where to find them (community associations).”*

*“We are not informed about associations.”*

#### Acceptability

We set out to access community “acceptability of ownership” of a text messaging programme. Our findings led us to rethink the question and present it in a way that was more understandable to participants. For this reason the discussion was presented as “willingness to participate” in a text messaging programme at any level. Opinions were favorable with regards to participating in a text messaging programme in six out of seven groups. They recognised the potential of such a programme in improving their adherence, quality of life and cohesion among people living with HIV.

*“People will join because they need more information on the illness, so they’ll come.”*

*“I will join if it will help my brothers and to sensitize others.”*

*“They will come if it’s free”*

One group unanimously decried the purpose of a text messaging programme. They urged for more self-management, as the text message would not affect the main components of care which they stated as having to come to the hospital and experience problems such as long wait times.

*“I am not in support”*

*“Really, I am indifferent.”*

*“I think that with this project, patients will no longer make any efforts”*

The participants who thought favorably of text messaging proposed that it could be used as a reminder for medication and appointments, to explain lab tests, as a source of solace, to provide general information (nutritional and educative) and as a social tool. They also expressed the need for an interactive service in which users can contact health workers for specific requests.

“*I want to be reminded of the time to take my medication.”*

*“…to deal with individual problems.”*

*“I need psychosocial support.”*

*“I should be told when medication is available at the pharmacy”*

*“We want to be able to call in case of specific problems.”*

However, they identified a number of challenges with the management of a text messaging programme such as limited financing, power outages (that would prevent them from charging their phones), the risk of accidental disclosure of status, limited organisational skills in associations of people living with HIV, lack of expertise in technological issues, phone network issues, tension between patients, illiteracy, and no clearly outlined benefits to participation in the programme.

“*We don’t have the financial means.”*

*“I think these text messages would not be useful to people who did not go to school.”*

*“…mismanagement by the leaders of the association.”*

*“We don’t have network (mobile phone signal) where I live.”*

#### Community readiness

Discussions about community readiness for ownership highlighted concerns about the resources required to sustain the programme. The participants expressed mixed feelings with regards to sources of financing and provided a list of potential funders: the state, para-public companies, private companies, non-governmental organisations and patients themselves.

*“The persons who had the idea should finance the project.”*

*“It’s the state……”*

*“It depends on my income.”*

*“I can contribute.”*

They felt that despite community involvement driven mostly by information needs, management by health personnel and social workers would be a valuable asset.

*“They (health personnel) are better informed than we are so…”*

*“They (health personnel) have a big role to play.”*

They also identified a number of factors that would impede the implementation of such a programme such as cost, the involvement of people who are not infected with HIV, the lack of integration with regular hospital services and the potential for disclosure of status.

*“People who are not sick may cause us trouble.”*

*“It will be very difficult for this community.”*

*“… Limited financing of the SMS”*

*“…shame of being recognised by others.”*

They were, however, optimistic about voluntarism and were willing to be involved.

*“I think there are always volunteers who are devoted to the care of patients.”*

*“We will try and see.”*

### Quantitative results

#### Participants

Four hundred and twenty participants (420) responded to the survey. Contrary to the qualitative sample, more men participated in the surveys (71.2%). Half of the participants had a secondary education (54.5) and two-thirds were employed (67.7%). The mean duration on antiretroviral therapy was 43.2 months (standard deviation [SD] = 37.06). The rest of their socio-demographic and clinical characteristics are displayed in Table 1.

**Table 1:** **Socio-demographic characteristics of 420 participants in the quantitative phase of the study**

Variable	Statistic
**Age (years): mean (SD)**	39.7 (10.28)
**Gender &: n (%)**	
Male	299 (71.2)
Female	118 (28.1)
**Level of education: n (%)**	
None	8 (1.9)
Primary	117 (27.9)
Secondary	229 (54.5)
University	66 (15.7)
Occupation: n (%)*	
Working	284 (67.6)
Unemployed	101 (24.0)
Retired	16 (3.8)
Student	15 (3.6)
**Marital status: n (%)**	
Married or living together	185 (44.0)
Single	169 (40.2)
Divorced	16 (3.8)
Widow or widower	50 (11.9)
**Residence: n (%)**	
Urban	344 (81.9)
Rural	76 (18.1)
**Lodging: n (%)** ^**#**^	
Common	357 (85.0)
Individual	56 (13.3)
**Time since diagnosis (months): mean (SD)**	57.2 (43.94)
**Duration on ARV (months): mean (SD)**	43.2 (37.06)
**Adherence to medication (7 point scale): mean (SD)** ^**#**^	5.6 (2.55)
**Adherence to appointments (7 point scale): mean (SD)** ^**$**^	6.0 (2.20)

^&^3 missing; *4 missing; ^$^6 missing; ^#^7 missing.

Data on the practical functioning of a text-messaging programme were also collected. Responses to these questions are summarised in Additional file 1.

#### Factors associated with current participation in community activities

Only 6.7% (n = 28) of the participants were currently involved in any community support programme. Only primary education (OR 30.15; 95% CI 1.88-483.15; p = 0.016) was associated with current participation in a community support programme. The Hosmer and Lemeshow goodness-of-fit test was as follows: chi-squared 3.26, df = 9, p = 0.833.

#### Factors associated with willingness to participate (acceptability) in a text messaging programme

Eighty percent (n = 336) of the participants expressed willingness to join a text messaging programme. Higher self-reported adherence to ART was associated with higher willingness to participate (OR 1.57; 95% CI 1.16-2.14; p = 0.004) and higher duration on ART was weakly associated with less willingness to participate (OR 0.95; 95% CI 0.92-1.00; p = 0.049). In addition, those who were not willing to pay for the text messaging were less willing to participate (OR 0.03 95% CI 0.00-0.28; p = 0.003). The Hosmer and Lemeshow goodness-of-fit test was as follows: chi-squared 7.188, df = 8, p = 0.519.

#### Factors associated with positive feelings (readiness) about community management

Three quarters, 73.6% (n = 309) thought the community could run the programme in a sustainable way and two-thirds, 61.7% (n = 259) were willing to participate in the development of the programme. Again participants who were less willing to pay where less likely to believe the community could run the project (OR 0.35; 95% CI 0.18-0.68; p = 0.002). The Hosmer and Lemeshow goodness-of-fit test was as follows: chi-squared 13.14, df = 8, p = 0.107.

#### Challenges to programme

Participants were able to identify a number of challenges that the community would face in setting up a text messaging programme. They reported lack of motivation among stakeholders (24.5%); limited funding (60.5%); participants phones out of order (25.2%); irregular phone network (38.6%); power outages (25.5%); fragile community organisations (6.9%); poor management from the PLHIV (36.9%); poor management from health personnel (32.9%); and limited experience in the implementation of the programme (13.3%).

#### Proposed measures

They proposed that PLHIV be involved in the management of the programme (27.6%); the programme should be based in the YCHATC (15.0%); ensure participation of health workers (54.0%); any communication should be coded (60.7%); and that messages should be personalised (25.2%).

#### Potential limitations

Illiteracy among PLHIV was the most frequently cited limitation (58.8%). Other problems cited were: financing (7.8%), non-ownership of phones (1.4%), language of the message (0.2%), inability to manipulate text messaging function (0.2%); lack of information on the programme (0.7%); lack of satisfaction with programme (0.2%); slow responses to participants’ queries (0.2%); medication stock-outs (0.2) and the stigma associated with HIV (0.2%).

### Data integration

**Figure 1:**
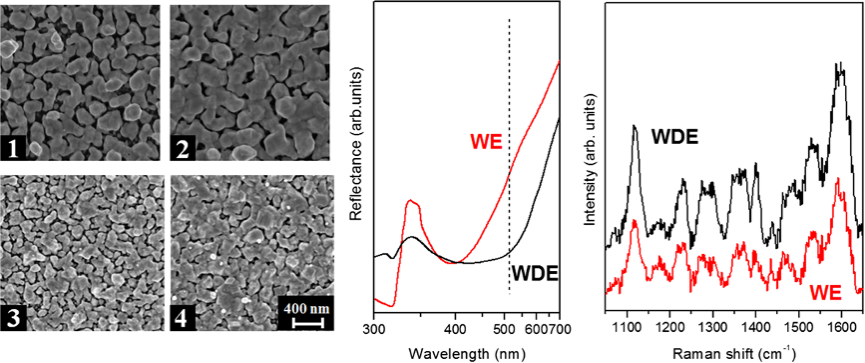
**Graphical display of merged data.**

A data matrix is presented in Table 2. The characteristics of the participants in both strands differed, with more urban dwellers in the quantitative strand, more females in the qualitative strand and more participants with a secondary or higher level of education in the quantitative strand. There was convergence in willingness to adhere to a text messaging project (acceptability) and the belief that the community could run a text messaging programme in a sustainable way (readiness). Similar (small) numbers confirmed current participation in community support programmes. Data regarding willingness to pay, participation of health workers and where the programme should be based did not converge See Figure 1.

**Table 2:** **Mixed-methods data matrix**

Strand							
Qualitative		Quantitative					
Themes identified	Number of times mentioned n/N (%)	Thematic variable	Representativeness n/N (%)	Influence on acceptability		Influence on readiness	
				OR (95% CI); p	aOR (95% CI); p	OR (95% CI); p	aOR (95% CI); p
*Acceptability*	61/67 (91.0)	*Would adhere to an SMS support initiative*	344/420 (81.9)	–	–	–	–
*Readiness*	63/80 (78.7)	*Believes community can run programme in a sustainable way*	295/420 (73.6)	–	–	–	–
*Acceptable cost*	175/210 (83.3)	*Willing to pay to receive SMS*	118/417 (34.4)	0.09 (0.01-0.68); 0.020	0.03 (0.00-0.29)	0.45 (0.26-0.80); 0.006	0.35 (0.18-0.66); 0.002
*Affinity to community projects*	6/96 (6.6)	*Currently participates in a community support initiative*	57.2 (43.94)	0 (0.0-0.0); 0.998	0 (0.0-0.0); 0.998	0.99 (0.41-2.40); 0.982	1.09 (0.41-2.97); 0.855
*Independent management*	58/67 (86.5)	*Prefers participation of health personnel*	227/417 (54.4)	0.92 (0.56-1.56);0.751	0.84 (0.49-1.43); 0.841	1.12 (0.72-1.75); 0.613	1.26 (0.77-2.02); 0.344
*Independent management*	45/67 (67.1)	*Prefers project be based in hospital*	63/419 (15.0)	0.79 (0.38-1.65); 0.543	0.83 (0.37-1.84); 0.648	1.17 (0.97-3.08); 0.060	1.62 (0.85-3.10); 0.142

n = numerator; N = denominator (derived from total of ideas raised); OR = odds ratio; aOR = adjusted odd ratio.

## Discussion

In this paper, we have explored the willingness of people living with HIV at the YCHATC to participate in a community led text messaging support programme, and their readiness. Overall, PLHIV would adhere to such an initiative and believe they are capable of managing the programme, with some reserve. Choices regarding how the initiative should be financed, where the programme should be based and how much health worker participation is desirable were areas of relative incertitude.

In our protocol we sought to investigate acceptability and readiness, however, in the course of our research the questions changed somewhat. Acceptability was better conceptualised as willingness to participate. Also, in our discussions about “readiness”, participants perceived readiness not only as having the skills and resources to manage a programme, but rather expressed positive or negative feelings with respect to community management. Given, the small numbers of participants who were already involved in a community programme (less than 8% in both strands), it is possible that many participants had no prior anchor which they could use to gauge readiness, and sought to conceptualise it in their own way.

We noted large differences in the composition of the participants in the qualitative and quantitative strands, notably with regards to gender and level of education. The qualitative strand (85.9% were females) seemed to reflect the gender distributions of people living with HIV who receive care at the YCH-ATC and who participate in text messaging studies (close to two-thirds were females) [36]. The inverse was seen in the quantitative phase (only 28.1%). However, these differences did not seem to affect acceptability and readiness as gender was not found to be an explanatory variable in the regression analyses. Also there were more participants with primary education in the qualitative strand (54.3%) compared to the quantitative strand (27.9%). Primary education was associated with current participation in community projects, even though very few participants in either strand participated in any community projects.

We also developed an innovative way of integrating qualitative and quantitative data, using forest plots. By estimating the confidence intervals around the point estimate, we are able to visually explore the extent of data convergence or divergence when the point estimates and sample sizes differ in the qualitative and quantitative strands. The confidence intervals give us a sense of the precision in our estimates coming from two samples with different sizes. However, these confidence intervals are estimated only for graphical purposes and should not be used for statistical inference. We used them to objectively infer convergence when there is overlap in the confidence intervals.

### Addressing the research questions

Acceptability of ownership of a text messaging programme is strong among YCHATC, but readiness as perceived by both researchers and participants demands that a number of issues be addressed. The main impediments to readiness were a lack of management skills and finances. Implementation of community ownership would therefore require training, maintaining a strong managerial body, and ensuring sustainable sources of funding, some of which were cited in the interviews.

The main limitation, with regards to implementation was the inability of some participants to read text messages. This implies that messages will have to be conceived in the plainest possible language. Another option worth considering is having participants use confidants to read the messages. Up to 43.4% reported having people who could receive and communicate text messages to them. The qualitative and quantitative strands were similar with regards to willingness to participate and readiness, but differed on where the programme should be based and whether health workers should be involved. For issues on which community opinions are divergent, feasibility and cost should be considered.

### Factors associated with acceptability and readiness

Participants with higher self-reported adherence to ART were more likely to find a text messaging project acceptable. This may reflect their understanding of the importance of high levels of adherence. On the other hand participants with a higher duration on ART were not willing to participate. Patient who have received ART for long periods may have developed other adherence enhancing strategies and would not be willing to include yet another [39]. They may also have developed hard-to-change medication taking habits. However, adherence to long term medication is known to wane over time and such patients require additional support [5, 39]. For such patients it is necessary to build on the other benefits to be reaped from such a project. Participants who were willing to pay for a text messaging project were more likely to be willing to participate and to be ready to own the project. Reducing the cost of the project, or increasing the value for money by augmenting the services provided may help in reaching participants who are less willing to pay.

### Explaining the lack of convergence

Less participants were willing to pay for a text messaging project in the quantitative strand (34.4%) compared to the qualitative strand (83.3%). This difference may reflect the flexibility in data collection used in the qualitative strand, with more opportunity for participants to discuss and consider different payment options, as opposed to the rigid closed-ended yes-no type questions in the quantitative strand. This also highlights the complementarity of both methodologies in eliciting a variety of responses for the same question. In the qualitative strand, more people wanted (86.5%) participation of health personnel than in the quantitative strand (54.4%). This divergence would suggest that there are unknown reasons why health worker participation may be perceived differently depending on how data were collected and should be investigated further. Some amount of health personnel participation is necessary to link participants to care, however their role as managers in a community led project is questionable. Previous research suggests high acceptability among health workers to take part in a text messaging programme despite the extra work involved [20]. The way forward would be to involve health workers only when necessary, by providing a permanent go-to person in the health facility. There was much more divergence with regards to where the project should be based, with 67.1% in the qualitative versus 15.0% in the quantitative strand proposing that it should be hospital-based. This may be a reflection of the expected participation of health workers, as health workers may be expected to participate more if the programme is hospital-based. It may also indicate the amount of independence that community members expect from the programme. A hospital based intervention holds numerous advantages in this context. Firstly, the hospital is the only common ground for all the PLHIV since they reside in various locations around the city of Yaoundé and it will be challenging to choose a convenient community location. Secondly, in the hospital it is easier to link the enhanced communication services of the text messaging programme with medical care and laboratory tests. Thirdly, the programme will benefit from the hospital infrastructure (locale, security, and internet). For smaller rural hard-to-reach communities, an outreach station may be beneficial. Such options warrant further consideration.

### Influence of context and researchers

Our findings should be interpreted as being relevant to the context of this health facility which in the previous years has been used for text messaging research among people living with HIV [18, 20, 32]. This paper builds on our previous findings and explores scaling up text messaging by letting the community own the programme. We cannot assume that participants were entirely naïve to the notions of text messaging and their responses in both the qualitative and quantitative strands may have been influenced by previous research activities. These findings are also tied to socio-economic, cultural and political factors which are specific to Cameroon.

### Diffusion of innovation

Within the theoretical framework of the diffusion of innovations theory, we have identified a number of innovation factors that will guide our framework development. Table 3 is a summary of these factors as they relate to community ownership of a text messaging programme.

**Table 3:** **Diffusion of innovation as applied to community ownership of a text messaging project**

Component of theory	Application to community ownership of a text messaging project
**Relative advantages to be gained**	Improved adherence to medication and appointment, assistance with pharmacy refills, psycho-social support, improved cohesion among PLHIV
**Compatibility with existing practices**	Weak participation in community activities, text messaging widely used for other purposes
**Simplicity of use, trialability and observable results**	Non-ubiquitous phone ownership and literacy, high trialability (opting out always possible), results can be appreciated by users
**Receiver factors (attitude to change and perceived need)**	High acceptability and perceived need
**Social system factors (social norms and tolerance for deviancy)**	Highly flexible system, positive health worker support

#### Framework development

**Figure 2:**
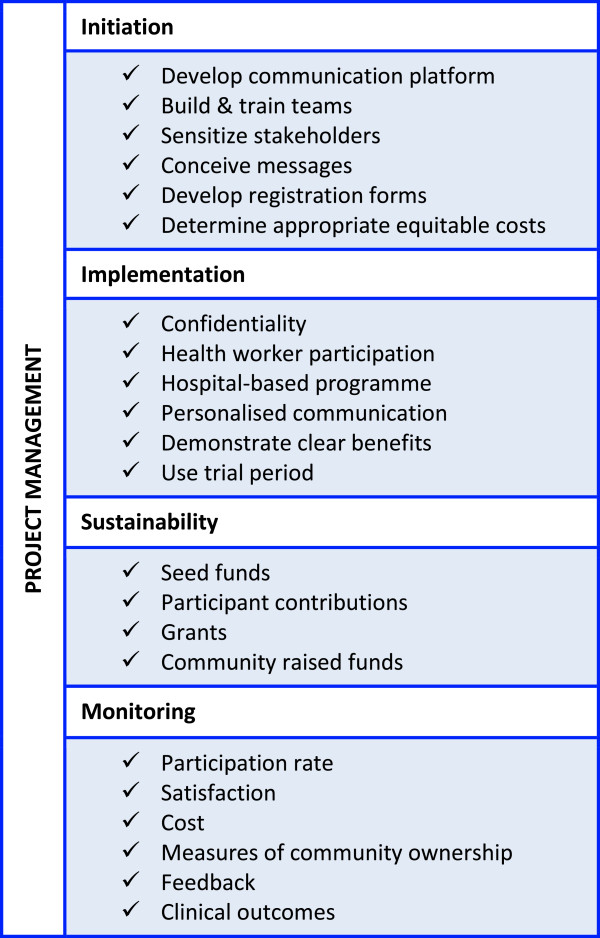
**Framework for community ownership of text messaging programme.**

Based on our findings in the qualitative and quantitative strands, we propose the following guidelines/framework for initiating community ownership of a text messaging programme:

- Incorporate health worker participation.
- Location may be community or hospital based (hospital based-preferable).
- Add other benefits to text messaging: provide other types of information, assistance with appointments, lab tests, picking up medication etc.
- Establish participation costs with potential participants.
- Provide a free test period.
- Personalize text messages as much as feasible.
- Build on community confidence – work with people they know and trust.
- Ensure high levels of confidentiality (non-explicit or encrypted messages).
- Publicize, take time to inform clients of new service
- Train community participants on relevant issues: confidentiality, programme management.
- Outline clear benefits for users.This guidelines should be applied in a cyclical manner, dropping off the less useful aspects and building on the strengths of the successful ones. Figure 2 is an illustration of this framework.

#### Strengths and limitations

The strengths of this study include the use of both qualitative and quantitative methodologies to elicit community acceptability and readiness of a text messaging programme, and employing cross-paradigm checks to ensure both internal and external validity.

Some limitations do exist, such as the lack of convergence in some variables and the fact that we have no direct measure of socio-economic status. A measure of socio-economic status would have been useful to bring more meaning to the data on acceptable cost.

## Conclusions

Our findings suggest that it is possible to initiate a community-owned text messaging project for PLHIV in Yaoundé, Cameroon. Community ownership is acceptable and potential users are willing to participate. Such a project can be developed by considering user-perspectives and needs, promoting capacity building in community project management, strengthening user confidence and developing a sound sustainable funding mechanism. This could be an important option for scaling-up text-messaging interventions that empowers the community and doesn’t weight on the limited human resources for health.

## Electronic supplementary material

Additional file 1: **Practical functioning of a text messaging programme.** (DOCX 13 KB)

## Abbreviations

ART: Antiretroviral therapy
CDBPH: Centre for the Development of Best Practices in Health
CI: Confidence interval
HIV: Human immunodeficiency virus
IEC: Information education communication
IRB: Institutional Review Board
OR: Odds ratio
PLHIV: People living With HIV
SD: Standard deviation
YCHATC: Yaoundé Central Hospital Accredited Treatment Centre.
