# Supplementary material for: A framework for community ownership of a text messaging programme to improve adherence to antiretroviral therapy and client-provider communication: a mixed methods study
Source: BMC Health Serv Res. 2014 Sep 26;14:441. doi: 10.1186/1472-6963-14-441 (PMC4263054; doi:10.1186/1472-6963-14-441)
Supplement: Supplementary file 1 — Additional file 1: Practical functioning of a text messaging programme. (DOCX 13 KB) [file 12913_2014_3528_MOESM1_ESM.docx]

**Additional file 1: Practical functioning of a text messaging programme**

| **Question** | **N (%)** |
| --- | --- |
| **For what reasons would you like to receive a text message?** | **333** |
| Medication reminders | 121 (36.4) |
| Appointment reminder | 84 (25.2) |
| Assistance with drug refill | 68 (20.4) |
| Assistance with lab test | 28 (8.4) |
| Others (psychosocial support, nutritional counselling) | 32 (9.6) |
| **Is there someone else who can receive the message and transmit it to you?** | **351** |
| Yes | 154 (43.8) |
| **How often would you be willing to pay to receive text messages?** | **143** |
| Monthly | 83 (58.0) |
| Quarterly | 42 (29.4) |
| Yearly | 18 (12.6) |
| **How should fees be charged?** | **138** |
| Flat rate | 96 (69.6) |
| Number of text messages expected | 42 (30.4) |
| **How much are you willing to pay per month (Frs CFA*)?** | **136** |
| 0-200 | 70 (51.4) |
| 200-500 | 30 (22.1) |
| 500+ | 36 (26.5) |
| **Why would you not participate** | **345** |
| Risk of disclosure of status | 161 (46.7) |
| Inability to pay | 122 (35.4) |
| Lack of confidence in organisers | 55 (15.9) |
| Intervention is not useful | 7 (2.0) |
| **What can be done to make PLHIV adhere to the programme?** | **414** |
| Sensitization | 209 (50.5) |
| Offer specific advantages | 121 (29.2) |
| Offer additional services | 84 (20.3) |
| **What kind of community organisation is best suited to run a text messaging programme?** | **314** |
| Non-governmental organisations | 150 (47.8) |
| Associations of PLHIV | 85 (27.1) |
| Health Committee | 77 (24.5) |
| Government | 2 (0.6) |
| **Would you advise a PLHIV to join a text messaging programme** | **413** |
| Yes | **382 (92.5)** |

*1 USD= ~500Frs CFA (minimum wage~59 USD)
